# Caudal Fossa Ratio in Normal Dogs and Eurasier Dogs with VLDLR-Associated Genetic Cerebellar Hypoplasia

**DOI:** 10.3389/fvets.2017.00241

**Published:** 2018-01-22

**Authors:** Alexander Lauda, Andreas Bruehschwein, Joanna Ficek, Martin J. Schmidt, André Klima, Andrea Meyer-Lindenberg, Andrea Fischer

**Affiliations:** ^1^Centre for Clinical Veterinary Medicine, Clinic of Small Animal Medicine, LMU Munich, Munich, Germany; ^2^Centre for Clinical Veterinary Medicine, Clinic of Small Animal Surgery and Reproduction, LMU Munich, Munich, Germany; ^3^Statistical Consulting Unit StaBLab, Department of Statistics, LMU Munich, Munich, Germany; ^4^Department of Veterinary Clinical Science, Small Animal Clinic, Justus-Liebig-University, Giessen, Germany

**Keywords:** VLDLR, genetic, Dandy–Walker malformation, cerebellar hypoplasia, posterior fossa, animal model, neuroimaging

## Abstract

Cerebellar and hindbrain malformations, such as cerebellar hypoplasia (CH), vermis hypoplasia, and Dandy–Walker malformation, occur in dogs as well as in humans. Neuroimaging is essential for a precise description of these malformations and defining translational animal models. Neuroimaging is increasingly performed in puppies, but there is a lack of data on developmental changes in the caudal fossa, which can impair assessment of caudal fossa size in this age group. The purpose of this study was to validate caudal fossa ratio (CFR) in dogs and to explore CFR in Eurasier dogs with genetic CH. CFR was calculated from midsagittal brain images of 130 dogs as caudal fossa area/total cranial cavity area. In addition, the volume of the caudal fossa was measured in 64 randomly selected dogs from this group. Repeated measurements were used to investigate inter- and intra-rater variability and influence of imaging modality. Furthermore, the influence of age, weight, and breed was explored. The CFR was a reliable parameter with negligible influence from the examiners, imaging modality, and weight of the dog. The midsagittal area of the caudal fossa and the volume of the caudal fossa correlated closely with each other. In this study, we observed a smaller CFR in puppies. The CFR in adult dogs lies within 0.255 and 0.330, while CFR is smaller in puppies up to 4 months of age. Besides age, there was also an effect of breed, which should be explored in larger data sets. Measurements of CFR in Eurasier dogs with genetic CH caused by a mutation in the very-low-density-lipoprotein-receptor gene revealed the presence of two variants, one with an enlarged caudal fossa and one with a normal to small caudal fossa. This observation indicates that there is phenotypic heterogeneity and interaction between the developing cerebellum and the surrounding mesenchyme in this animal model.

## Introduction

Animal models of human disease provide insights into pathophysiology at a molecular level, and gene discovery in dogs has become an important resource. In human medicine, neuroimaging techniques have helped to improve the definitions of hindbrain and cerebellar malformations. The classification of midbrain, cerebellar, and hindbrain malformations has evolved from several image-based classification systems to a classification system that is based on embryonic development (1–4). This system was produced by modern neuroimaging techniques as well as increased understanding of correlations between gene mutations and certain brain imaging phenotypes. The recognition of phenotypic heterogeneity of certain gene mutations and the fact that different gene mutations may result in nearly identical neuroimaging findings has further contributed to the understanding of these mutations (4, 5). Many of these data are derived from animal studies (2).

**Figure 1:**
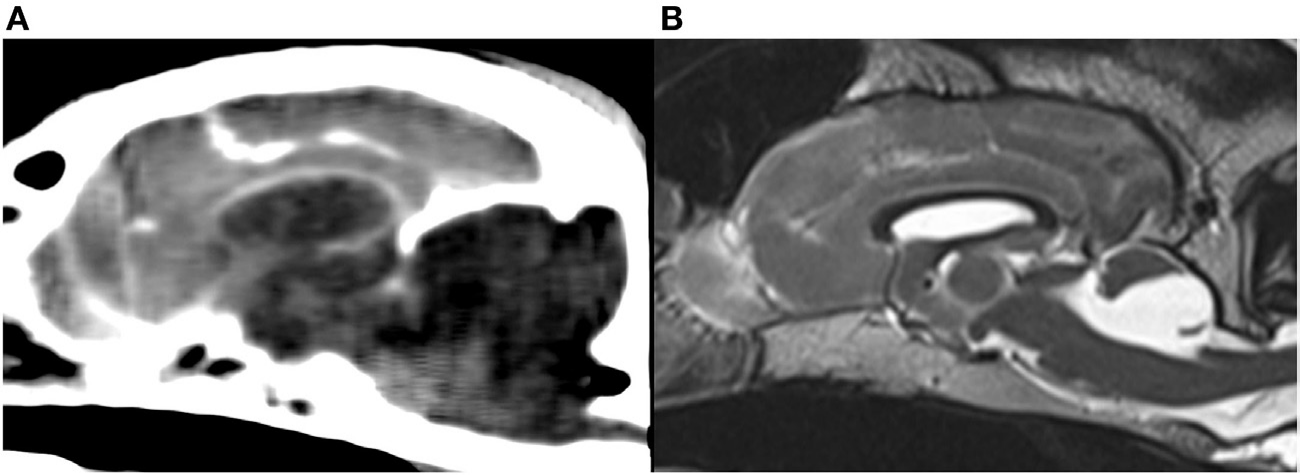
Midsagittal brain images of Eurasier dogs with *VLDLR*-associated cerebellar hypoplasia. **(A)** CT, soft tissue window, large fluid accumulations within caudal fossa and subjectively enlarged caudal fossa, **(B)** T2-weighted MRI, retrocerebellar fluid accumulations with a cyst-like appearance and subjectively normal to small caudal fossa. Panel **(B)** was originally published under (7). Reuse is permitted by the Creative Commons Attribution CC BY license.

Genetic cerebellar hypoplasia (CH) in Eurasier dogs represents the first genetically defined CH that has been described in dogs. It is caused by a fully penetrant recessive mutation in the very-low-density-lipoprotein-receptor gene (*VLDLR*) (6, 7). Genetic testing for breeders is now available (6, 7). The VLDLR is part of the Reelin signaling pathway and influences cell migration and positioning during embryonic development, especially in the cerebellum (8–10). The neuroanatomic changes are absence of the caudal aspects of the cerebellar vermis and the cerebellar hemispheres in association with large retrocerebellar fluid accumulations (11, 12). Initial observations suggested an abnormal configuration of the caudal (cranial) fossa and an enlarged caudal fossa as an additional imaging feature in some puppies with *VLDLR*-associated CH resembling a Dandy–Walker like malformation [Figure 1; reported in Table 3 of Ref. (7)]. The evaluation was problematic, however, because of the lack of reference data in the literature related to caudal fossa size in puppies and the different imaging modalities used.

Therefore, the aim of this study was to validate the use of the caudal fossa ratio (CFR) from area measurements of midsagittal brain images to assess caudal fossa size in dogs of various age groups, body weights, and breeds. Specifically, we aimed to explore the application of CFR to puppies with and without *VLDLR*-associated CH to define the animal model in more detail.

## Materials and Methods

Caudal fossa ratio was calculated from midsagittal brain images of 130 dogs. Study design was retrospective and observational. The data of the dogs in this study were used with the consent of their owners and the breed club. The study was approved by the institutional research review board (#09-06-2014).

### Control Dogs (*n* = 111)

Magnetic resonance images from 111 control dogs (83 dogs > 6 months of age, 28 dogs ≤ 6 months of age) were derived from the MRI database of two institutions (Clinic of Small Animal Surgery and Reproduction, LMU Munich; Small Animal Clinic, Justus-Liebig-University, Giessen). Inclusion criteria were unremarkable MR images without any evidence for intracranial or skull disease. All dogs presented for neurological signs. The most common diagnosis was idiopathic epilepsy and geriatric vestibular syndrome. The adult dogs included 10 Australian Shepherds, 10 Border Collies, 13 Golden Retrievers, 14 Labrador Retrievers (large mesaticephalic breeds), 7 Boxers, 10 French Bulldogs, 7 Pugs (small and large brachycephalic breeds), and 12 Eurasier dogs without CH (Table 1). The breed and age of the puppies is outlined in Table S1 in Supplementary Material. The number of male and female dogs in each group is outlined in Table S2 in Supplementary Material. MRIs of the head were performed using a 1.5 T scanner (Magnetom Symphony Syngo MR, Siemens AG, Erlangen, Germany) or a 1.0 T scanner (Gyroscan Intera, Philips, Hamburg, Germany).

**Table 1:** Caudal fossa ratio (CFR) of Eurasier dogs with *VLDLR*-associated cerebellar hypoplasia and control dogs.

Group		Age (months)		CFR		
		Median	Range	Median	Range	Mean ± SD
Eurasier (variant 1)	*n* = 3	2	2–3	0.354	0.344–0.441	0.379 ± 0.044
Eurasier (variant 2)	*n* = 8	2	2–56	0.232	0.191–0.292	0.249 ± 0.050
**Control dogs > 6 months of age**						
Eurasier (unaffected)	*n* = 12	52.5	13–178	0.315	0.265–0.330	0.304 ± 0.021
**Mesaticephalic**						
Australian Shepherd	*n* = 10	52	20–130	0.299	0.285–0.320	0.299 ± 0.011
Border Collie	*n* = 10	44.5	13–87	0.286	0.258–0.303	0.286 ± 0.012
Golden Retriever	*n* = 13	111	45–163	0.287	0.277–0.319	0.291 ± 0.012
Labrador Retriever	*n* = 14	82.5	8–176	0.299	0.274–0.324	0.299 ± 0.013
Total	*n* = 47	71	8–176	0.291	0.258–0.324	0.294 ± 0.013
**Brachycephalic**						
Boxer	*n* = 7	93	9–119	0.273	0.261–0.283	0.275 ± 0.010
French Bulldog	*n* = 10	48	7–74	0.308	0.284–0.315	0.304 ± 0.010
Pug	*n* = 7	30	7–107	0.285	0.255–0.304	0.282 ± 0.018
Total	*n* = 24	55.5	7–119	0.291	0.255–0.315	0.289 ± 0.018
**Control dogs ≤ 6 months of age**						
Different breeds (<4 months)	*n* = 20	3	1–4	0.256	0.226–0.296	0.256 ± 0.018
Different breeds (4 < 6 months)	*n* = 8	6	5–6	0.287	0.256–0.304	0.279 ± 0.016

### Comparison between MRI and CT (*n* = 8)

Eight additional dogs of various breed and age were included. Both imaging modalities, MRI and CT, were available from these dogs. These dogs suffered from various intracranial diseases. MRIs were performed using a 1.5 T scanner (Magnetom Symphony Syngo MR, Siemens AG, Erlangen, Germany). CT scans of the head were obtained using a multislice CT (Somatom Definition AS, Siemens AG, Erlangen, Germany).

### Eurasier Dogs with CH (*n* = 11)

Eleven pure-breed Eurasier dogs with genetic *VLDLR*-associated CH (8 MRI, 3 CT) were included. The affected Eurasier dogs were between 2 and 7 months of age (median 2 months). One dog was an adult at the time of imaging (56 months).

### Morphometric Studies

**Figure 2:**
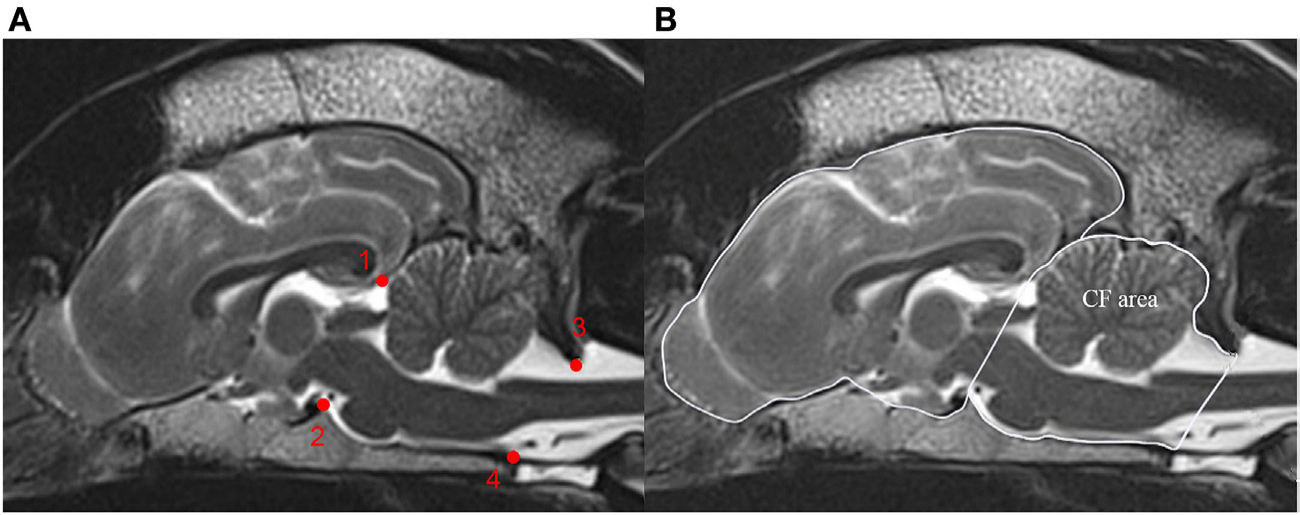
Calculation of the caudal fossa ratio (CFR) on midsagittal brain images. The figure shows midsagittal T2-weighted magnetic resonance images of the brain of a control dog. **(A)** Bony structures used to identify the boundary of the caudal fossa. (1) Most rostral aspect of the tentorium cerebellum, (2) dorsum sella turcica, (3) caudodorsal margin of the foramen magnum, and (4) ventral margin of the foramen magnum. **(B)** Measurements on midsagittal T2-weighted MRI figure displays outline of the caudal fossa (CF area) and total cranial cavity (peripheral boundary line) for area measurements. CFR is calculated as CF area/total cranial cavity area.

MRI measurements were conducted on T2-weighted midsagittal brain images. CT measurements were conducted on midsagittal brain images with soft tissue (window width, 300; window level, +30) and bone reconstruction algorithm (window width, 2,500; window level, +500). Five image series needed to be readjusted to obtain measurements in midsagittal planes. Measurements of the midsagittal areas of the cranial cavity case and of the caudal fossa (cm^2^) were based on manual delineation of the inner surface of the skull and calculations with conventional imaging software (Osirix^®^; v.5.6 Pixmeo Sarl). The cranial and caudal extensions of the caudal fossa were straight lines connecting the most rostral aspect of tentorium cerebellum to the dorsum sella turcica as well as the caudodorsal margin of the foramen magnum to the ventral margin of the foramen magnum (Figure 2A) (13). All measurements were performed twice and the mean was used for all calculations. The CFR reflected the relative size of the caudal fossa cross-sectional area and was calculated as the caudal fossa area/total cranial cavity area for all images (Figure 2B).

Furthermore, we made volumetric measurements of the caudal fossa from 64 randomly selected dogs from the control group with a second imaging software (3D Slicer^®^; v.4.4.0). The caudal fossa was defined as the space bound by the dorsal surface of the basioccipital bone, the dorsum sellae, the foramen magnum, and the apical part of the petrous part of the temporal and the basioccipital bones (14). The caudal fossa was manually delineated on each midsagittal and parasagittal T2-weighted MR image, and the caudal fossa volume was calculated by the software.

### Reliability

MRI scans of eight dogs from the control group were selected in a randomized manner for assessment of intra- and inter-rater reliability of CFR. Investigators were blinded to breed, age, and the results of previous measurements. Intra-rater variability was done by repeated measurements by the first author of the study (AL). In five dogs, CFR was measured once a day on five consecutive days (Monday to Friday) for 4 weeks; in three dogs CFR was measured once a week (Monday) for 6 weeks. Intra-rater variability was assessed by visual inspection of repeated measurements. Furthermore, the coefficient of variation was calculated to measure the dispersion of the measurements. For assessment of inter-rater variability, the same eight studies were evaluated by the first author of the study (AL) and then by a board-certified radiologist (AB) following instructions for an example case. Inter-rater variability was evaluated in linear regression. The association between caudal fossa area and caudal fossa volume was assessed in 64 dogs of the control group with linear regression analysis.

### Statistical Analysis

Exploratory analysis was conducted to investigate the influence of age, weight, and breed on CFR. Based on the study of Ref. (15), we decided to compare four age groups: 1–4 months, 5–6 months, 7–12 months, and >1 year (15). A one-way ANOVA was performed to assess the overall effect of age in control dogs, preceded by the Shapiro–Wilk test for normality. The null-hypothesis could not be rejected in any of the analyzed groups. In the performed tests, the level α = 0.05 was used to determine significance. The effect of weight was evaluated in linear regression. The difference between imaging modalities was examined in exploratory analysis and assessed in linear regression with the CT measurement ratio as the dependent variable and the MRI measurement ratio as the independent variable. In all the regression analysis in this paper, a simple regression model: *y*_i_ = β_0_ + β_1 _×_ _*x*_i_ + ε_i_ was used, where β_0_ stands for intercept and ε for the random error component. The regression coefficient β_1_ represents the effect of the independent variable *x* on the dependent variable y. Moreover, the square root with the sign of β_1_ of the *R*-squared is the linear correlation coefficient between *x* and *y*. In all tests, the level α = 0.05 was used to determine significance. All the statistical analyses were performed in R software version 3.3.1.

## Results

### Reliability

**Figure 3:**
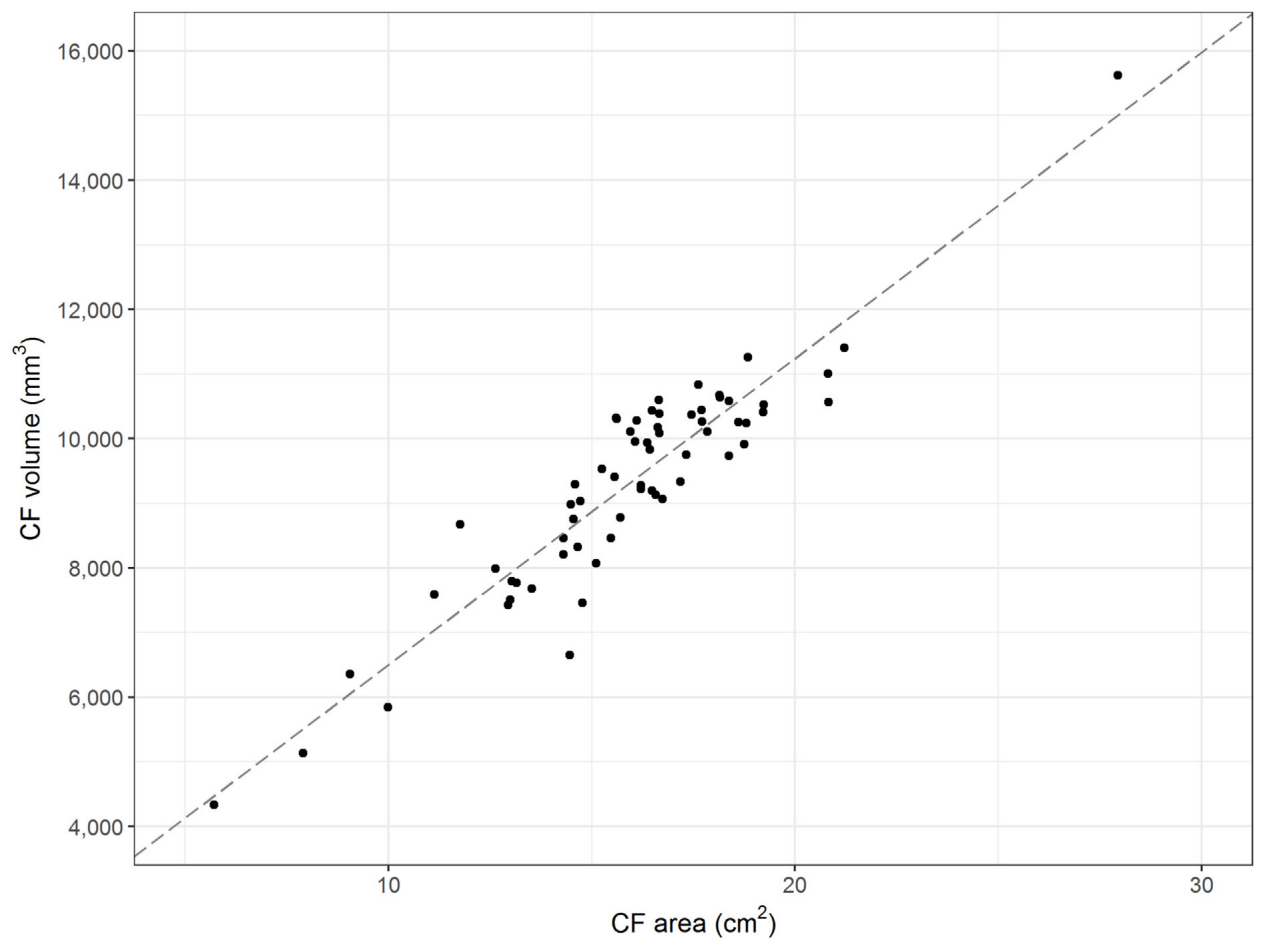
Midsagittal area and volume of the caudal fossa. The figure displays the midsagittal area of the caudal fossa (cm^2^; Osirix^®^) of 64 control dogs on the *x*-axis and the corresponding calculations of the volume of the caudal fossa (mm^3^; 3D Slicer^®^) on the *y*-axis. Each dot represents the measurements of one individual dog. The line represents the regression line and shows linear correlation between midsagittal area and volume (correlation coefficient 0.93; *R*-squared 0.86).

Data confirm the reliability of CFR as a robust parameter for assessment of caudal fossa size. The mean intra-rater coefficient of variation equals 0.0066. Visual inspection of the repeated measurements suggests no presence of learning effects. The regression performed to assess the inter-rater reliability implies that there was a minor effect of researcher performing measurements on the CFR. The correlation coefficient equals 0.95 (*R*-squared 0.91) and the regression coefficient (β_1_) is 0.91. Visual and statistical comparisons between area and volume measurements proved positive linear correlation between the midsagittal area and volume of the caudal fossa in dogs. The correlation coefficient equals 0.93 (*R*-squared 0.86) (Figure 3).

### Influence of Age

**Figure 4:**
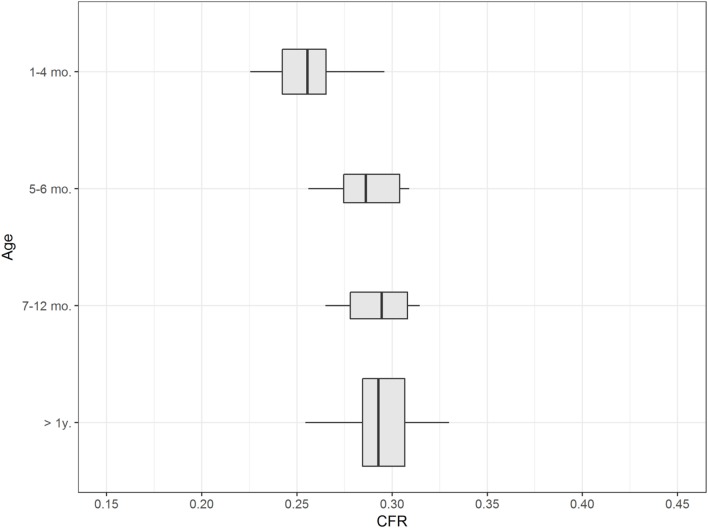
Influence of age. The figure shows box plots of the caudal fossa ratio (CFR) of dogs from the control group. Each box plot displays CFR from dogs from one age group: ≤4 months, 5–6 months, 7–12 months, and >1 year. The width of the box plots corresponds to the number of dogs in each age group. Dogs ≤ 4months of age show a relatively smaller CFR compared to older dogs.

Caudal fossa ratio was assessed in 111 control dogs of various ages. CFR was compared between four age groups: ≤4 months (*n* = 20), 5–6 months (*n* = 8), 7–12 months (*n* = 7), and >1 year (*n* = 76). All groups contained dogs with various skull morphologies. Age had a significant influence on CFR (*p* < 0.001; Table S3 in Supplementary Material). We observed a smaller CFR that was indicative of a relatively smaller size of the caudal fossa in cross-sectional images in puppies up to 4 months of age compared to adult dogs with completed skeletal growth (Figure 4; Table 1). CFR stabilized at 4 months of age and did not change after that point.

### Influence of Breed and Weight

**Figure 5:**
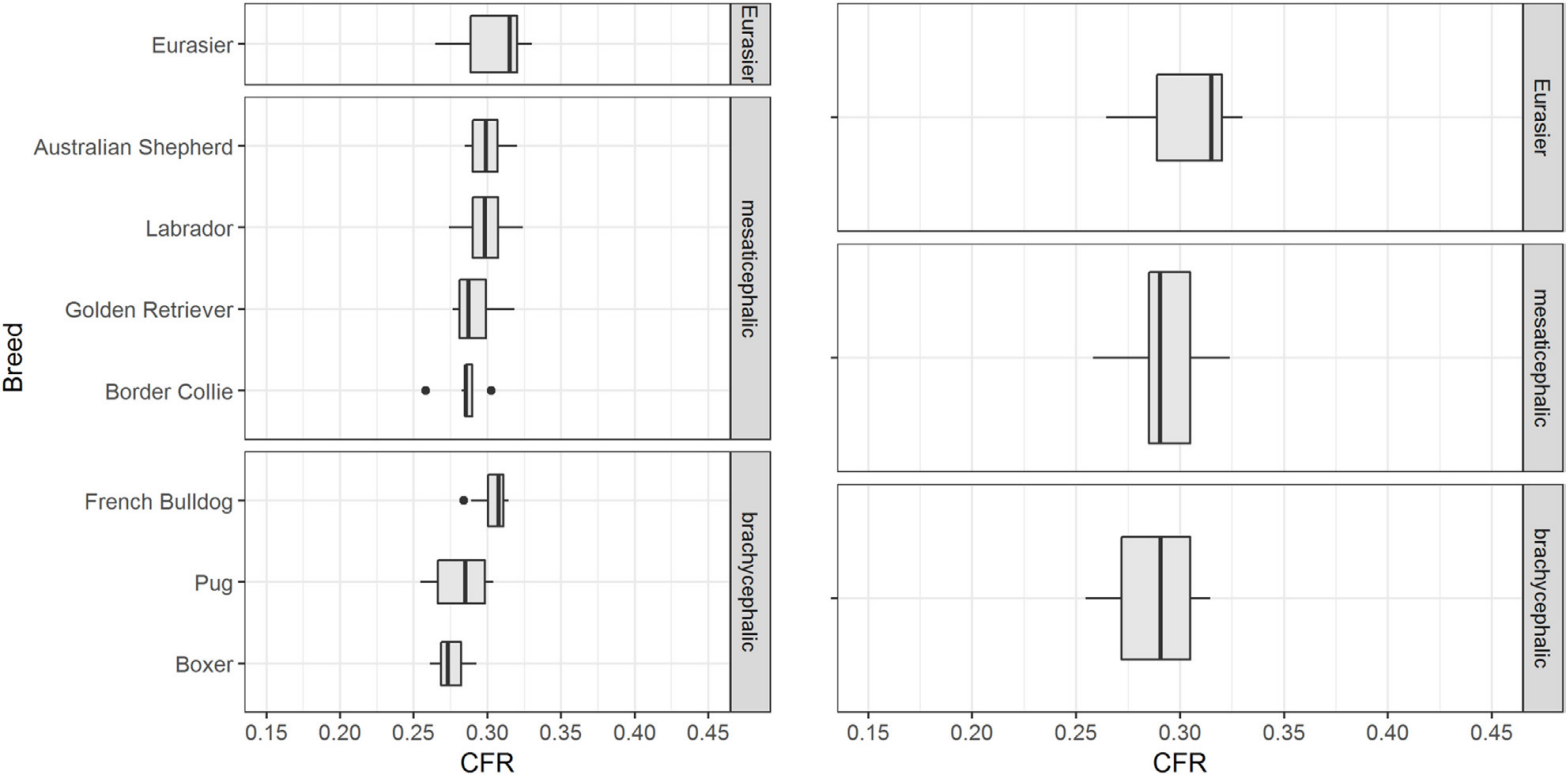
Influence of breed. The figure shows box plots of the caudal fossa ratio (CFR) from dogs of the control group. Each box plot displays CFR of dogs from one breed. There are visible differences in CFR between breeds, especially within the brachycephalic group.

**Figure 6:**
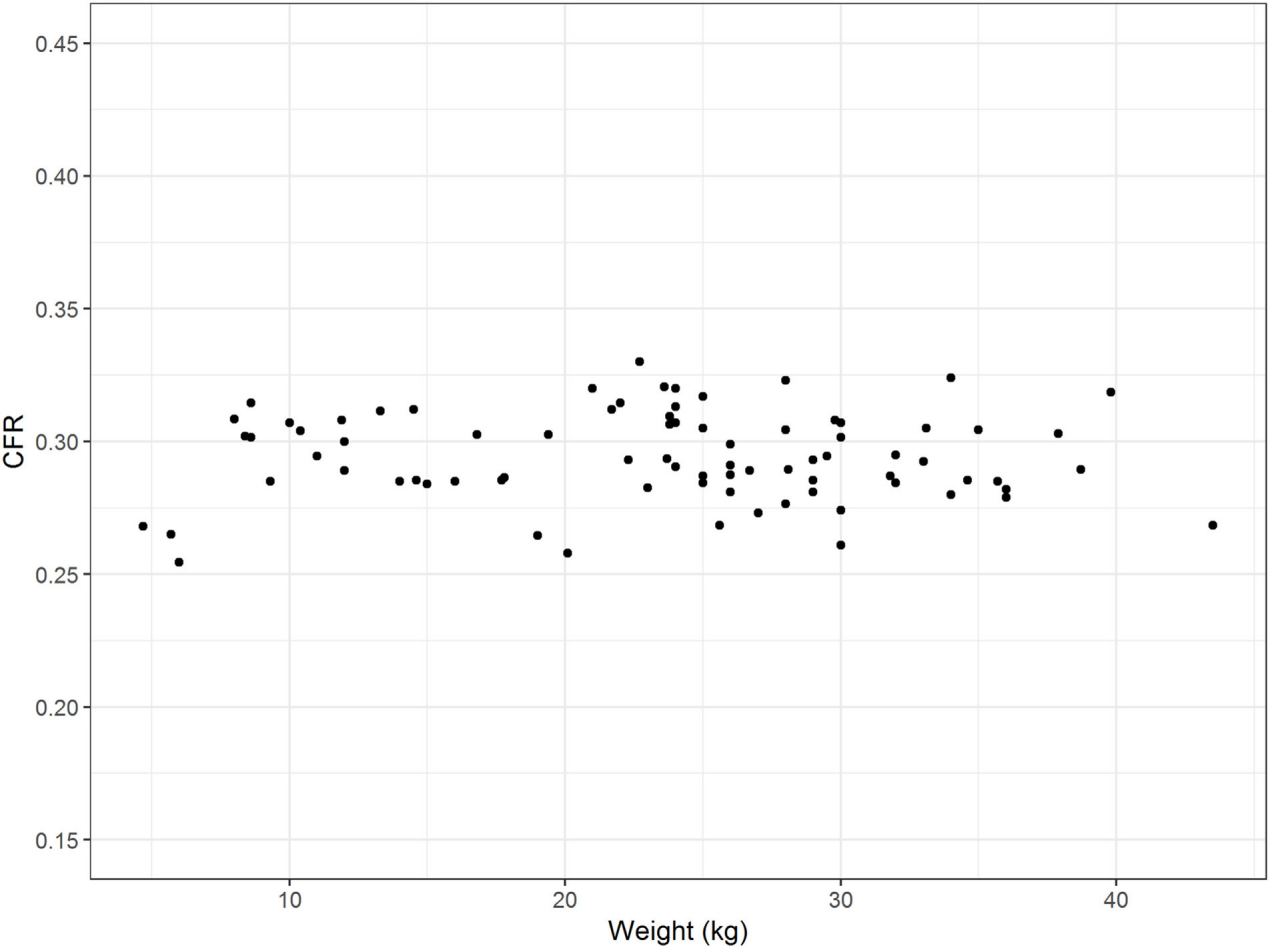
Influence of weight. The figure displays the weight (kilograms) of the dogs on the *x*-axis and the corresponding caudal fossa ratio (CFR) on the *y*-axis. Each dot represents the measurements of one individual dog from the control group. There is no association between weight and CFR.

The influence of breed on CFR was assessed in 83 control dogs ≥ 6 months of age of eight different breeds. CFR ranged from 0.255 to 0.330 (0.293 ± 0.015). There were visible differences in CFR between breeds, especially between the brachycephalic breeds (Figure 5). However, the effect of breed on CFR was not substantial compared to the effect of age. The linear regression estimator was close to 0 (β_1_ < 0.001) and therefore the effect of weight was negligible (Figure 6).

### Imaging Modality

**Figure 7:**
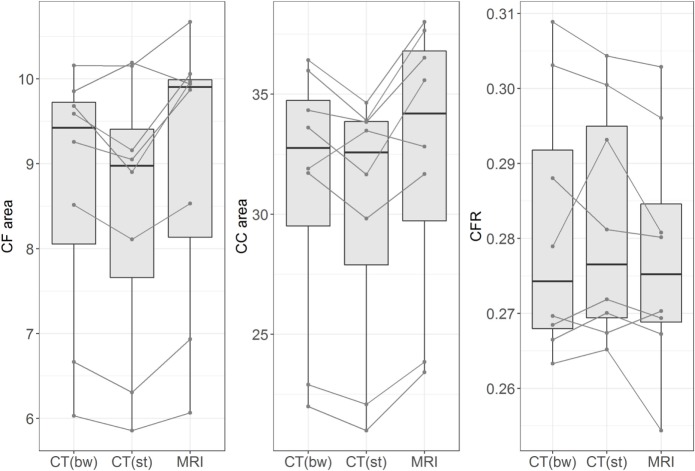
Influence of imaging method. The figure shows box plots of measurements of midsagittal caudal fossa area (CF area, left figure), cranial case area (CC area, middle figure), and caudal fossa ratio (CFR, right figure). Each figure displays the measurements from eight dogs with different imaging methods: CT (bw), computed tomography with bone window algorithm; CT (st), computed tomography with soft tissue window algorithm; MRI, T2-weighted magnetic resonance images. The lines connect the corresponding measurements from each dog. Midsagittal CF areas and CC areas are smaller in CT (bw) and CT (st) than in MRI, but this effect is no longer seen when CFR is compared between the three imaging modalities.

The differences in CFR between MRI and CT images (soft tissue window and bone window) were evaluated in eight dogs and were negligible. There was only a minor influence of the imaging modality on CFR (Figure 7). Measurements of the midsagittal area of the caudal fossa and the whole cranial area were smaller in CT than MRI, but this effect was no longer visible in the ratio. For the MRI ratio to CT ratio (soft tissue window) the correlation coefficient equals 0.95 (*R*-squared 0.90). The regression coefficient was 0.93 and thus, for one unit increase in MRI ratio, the CT ratio increases by 0.93 units. For the MRI ratio to CT ratio (bone window) the correlation coefficient equals 0.97 (*R*-squared 0.93 and regression coefficient 1.06). For the CT ratio (bone window) to CT ratio (soft tissue window) the correlation coefficient equals 0.93 (*R*-squared 0.86 and regression coefficient is 0.83).

### Eurasier Dogs with *VLDLR*-Associated CH

**Figure 8:**
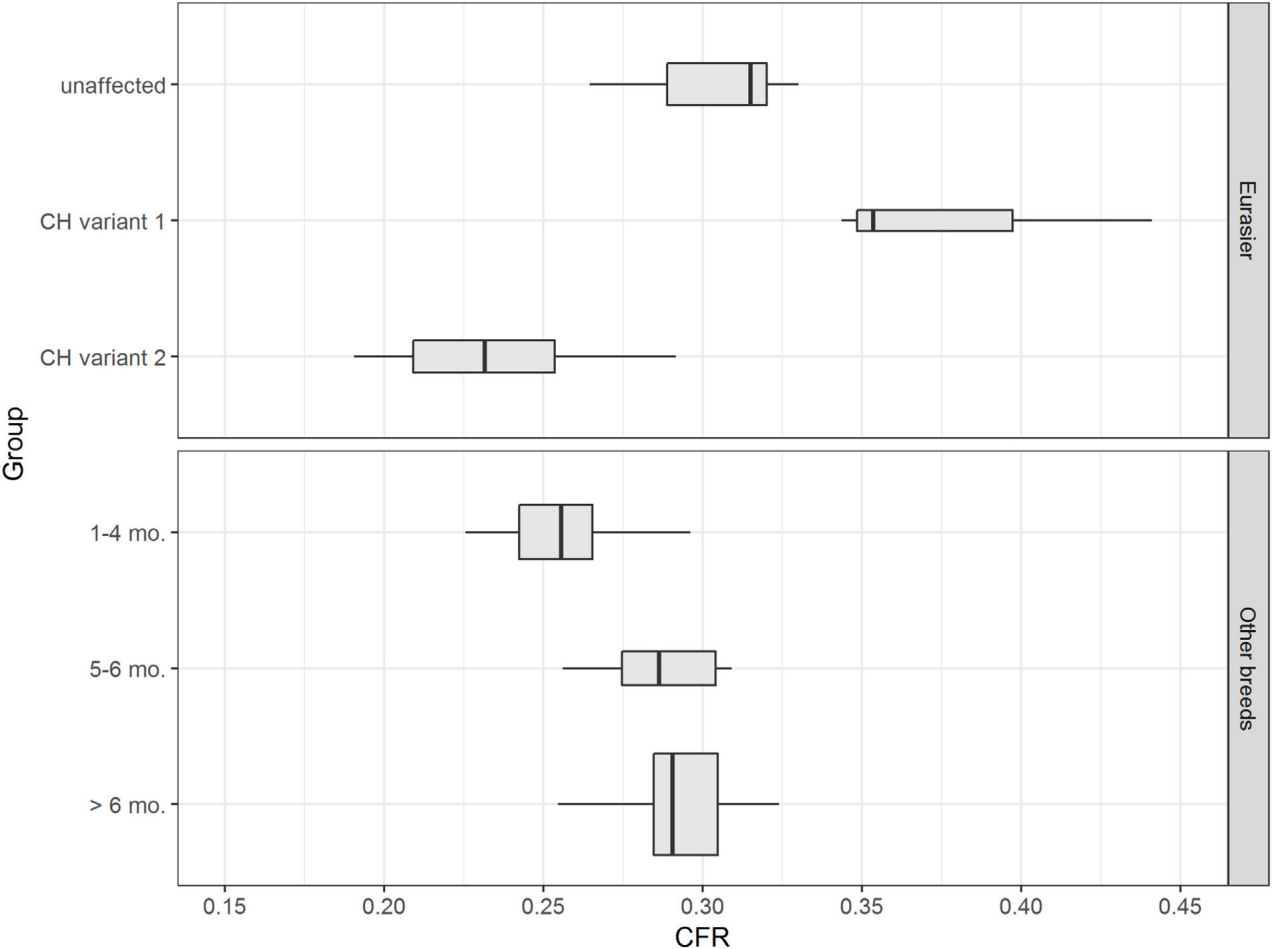
Eurasier dogs with *VLDLR*-associated cerebellar hypoplasia (CH). The upper figure displays boxplots of caudal fossa ratio (CFR) from unaffected Eurasier dogs and Eurasier dogs with CH. The lower figure shows box plots of CFR from control dogs. Measurements of CFR in Eurasier dogs with genetic CH demonstrated the presence of two variants, including variant 1 with an enlarged caudal fossa and variant 2 with a normal-to-smaller caudal fossa.

Data confirmed a wide variation in caudal fossa size in Eurasier dogs with *VLDLR*-associated CH. Assessments were based on measurements of CFR in Eurasier dog puppies with CH and comparisons made to puppies of other breeds, adult unaffected Eurasier dogs and adult dogs of other breeds (Figure 8). CFR was high and above the range observed in the control dogs, which indicated the presence of an enlarged caudal fossa in three dogs with genetic CH (variant 1). In the other dogs, CFR was at the lower limit or below the range observed in control dogs (variant 2) (Table 1). These results were further supported by a descriptive analysis (Figure S1 in Supplementary Material).

## Discussion

The caudal cranial fossa henceforth in this manuscript referred to as caudal fossa for ease of reading, which is called the posterior fossa in humans, is the inner surface of the skull that surrounds the brainstem and cerebellum. Its rostral and caudal borders are the tentorium cerebelli and the foramen magnum (13, 14). A variety of brainstem and cerebellar malformations are associated with disturbed growth of the cranium (2, 3). Consequently, assessment of caudal fossa size should be part of the description and definition of animal models of brain malformations. A precise description of congenital malformations enhances comparisons with analogous diseases in humans.

Our findings establish CFR as a reliable parameter for assessment of the relative size of the caudal fossa. The measurements demonstrate excellent agreement within and between raters (intra-, inter-rater agreement). There is only a minor influence of imaging modality on the ratio and results are in the same range as reported from other authors (13, 16).

An important finding is the lack of impact of weight on CFR (Figure 6). This finding supports the use of CFR as a parameter that is independent of the size of the dog and can be applied to large dog breeds as well as small dog breeds and over a wide range of weights. Dog breeds also differ according to skull morphology. A review of groups of dogs from different breeds indicated an influence of breed on CFR that should be addressed in future studies. While we failed to find overall influence of a brachycephalic or a mesaticephalic skull, there were visible differences in CFR between small and large brachycephalic breeds.

We postulate that area measurements are precise parameters and reflect the basic configuration and the size of the caudal fossa in relation to the whole skull. It has been stated that three-dimensional assessments (volume measurements) are more accurate for assessment of caudal fossa size than midsagittal area measurements in dogs (17). However, this statement occurred in the context of an investigation of pathomechanisms in Chiari malformation/syringomyelia in Cavalier King Charles Spaniels in which assessment of overcrowding requires precise measurements of the proportions of brain parenchyma and CSF space. Our investigations in 64 dogs with various skull conformations showed a close correlation between midsagittal caudal fossa area and caudal fossa volume. This result confirms the validity of CFR and provides further support for the use of CFR for the characterization of animal models. It should be noted, however, that previous studies in humans showed variable results and either weak (18) or no (17) correlation between area and volume measurements. Others have successfully applied a brainstem/cerebellar ratio for diagnosis of cerebellar atrophy and cerebellar degeneration in dogs (19). They also showed close correlation between area and volume measurements. Both brainstem/cerebellar ratio and CFR may be used for phenotyping cerebellar and hindbrain malformations in dogs.

Our data show that puppies up to 4 months (19 weeks) of age have a smaller CFR than adult dogs (Figure 4). This observation has not appeared in the literature to date. Some investigators excluded dogs younger than 12 months of age from measurements based on their assumption of an influence of incomplete skull growth (20, 21). Other researchers considered dogs younger than 12 months of age for their measurements (13, 22) or dogs as young as 4 months of age and assumed that there was no influence of skull growth on their results (23). Early diagnosis of brain malformations is advantageous to breeders and clients. Furthermore, neuroimaging of affected dogs and their littermates may be necessary for confirmation and precise definition of the phenotype for genetic investigations (7). A smaller CFR in puppies up to 4 months of age reflects discordant growth of the skull in puppies in which the growth of the caudal fossa is smaller. Disproportionate growth started at 50 days in German Shepherd puppies. Excessive growth of the caudodorsal cranium with development of the external sagittal crest and occipital protuberance occurred from days 70 to 107 (15).

We used CFR to validate caudal fossa size in an animal model of a genetic CH. A variant in the very-low density lipoprotein receptor gene (VLDLR:c.1713delC) is the cause of inherited CH in the Eurasier dog breed. Inheritance is recessive and genetic testing is now available for breeders to avoid the disease (6). Clinically, homozygous dogs present with non-progressive ataxia which is first evident when the animals start to walk. VLDLR is part of the Reelin signaling pathway and involved in neuronal migration and cerebellar development (8–10). The analogous human disease has been described as disequilibrium syndrome and involves mutations in *VLDLR*. To date, there have been 12 reports from 15 families and 45 involved persons (24–30). Both humans and dogs present non-progressive cerebellar (truncal) ataxia as the main complaint. Neuroanatomic changes are strikingly similar between the two species. Caudal aspects of the cerebellar vermis and the cerebellar hemispheres are absent and replaced by large retrocerebellar fluid accumulations. Our measurements of CFR in Eurasier dogs with *VLDLR*-associated CH and appropriate controls confirm the presence of two variants: variant 1 with a grossly enlarged caudal fossa and variant 2 with a small to normal-sized caudal fossa. This observation has several implications. First, it proves phenotypic variability with regard to the size of the caudal fossa in dogs with *VLDLR*-associated CH. Wide variability in the size of the posterior fossa from normal to large is also seen in humans and mice with *FOXC1*-associated Dandy–Walker and related malformations (5). Furthermore, our observations also support an interaction between the cerebellum and the overlying mesenchyme (meninges, skull) during development. The concept that cerebellar and posterior skull development are closely linked through interactions between the rhombic lip and the overlying mesenchyme (3, 31) arose from observations in *FOXC1* mice with Dandy–Walker malformations. These mice express *FOXC1* only in the mesenchyme overlying the cerebellum and not in the cerebellum itself (5). Finally, variability in genetic background and interaction between different genes could also contribute to the conformation of the caudal fossa in this dog breed. We observed slightly more variability in caudal fossa size in unaffected Eurasier dogs compared to other dog breeds. Finally, there could be an impact of environmental or vascular factors during development. Limitations of this study are obviously the lack of MRI from breed- and age-matched controls as well as absence of measurements on T1-weighted midsagittal images and that we combined images from a 1.5 and 1.0 T MR. Furthermore, the size of the breed groups was too small to asses the influence of breed and larger cohorts with more breeds representatives of their skull type and separating large and small brachycephalic dogs (and other skull types) into different control groups need to be examined. However, CFR in some pupies with vldlr-associated CH was even larger than in adult Eurasier dogs and clearly exceeded any of our control dogs.

In summary, our data show that CFR is a reliable parameter with a negligible influence of imaging modality, examiner, and weight of the dog and a minor influence of breed. The CFR in healthy adult dogs lies within 0.255 and 0.330, but CFR is smaller in puppies. Furthermore, the midsagittal area of the caudal fossa is positively correlated with caudal fossa volume. Measurements in Eurasier dog puppies with genetic *VLDLR*-associated CH demonstrated the presence of a variant with an enlarged caudal fossa. This observation further substantiates the application of CFR for characterization of translational animal models of cerebellar and hindbrain malformations.

## Ethics Statement

The study was carried out in accordance with the German Animal Protection law. The protocol was approved by the institutional research review board of the Clinic of Small Animal Medicine; #09-06-2014. Dog owners approved use of their dogs’ data for scientific purposes by signature in clinical records. Some images from diseased dogs were contributed by the breed club with the consent of the owners for research purposes.

## Author Contributions

AL, AF, AB, and ALM designed and coordinated the study. AL and AB determined the measurements. JF and AK performed the statistical analysis. MS contributed in acquisition of the data. AL, AF, and JF wrote the manuscript. All authors read and approved the final manuscript.

## Conflict of Interest Statement

The authors declare that the research was conducted in the absence of any commercial or financial relationships that could be construed as a potential conflict of interest.

## Supplementary Material

Figure S1 Exploratory data analysis of caudal fossa ratio (CFR) in Eurasier dogs. The plots depict the distribution of CFR in unaffected Eurasier dogs and Eurasier dogs with cerebellar hypoplasia (CH). To achieve better comparability, the CFR was adjusted for age. Measurements of unaffected dogs were centered according to age group. The mean of measurements of unaffected dogs from the corresponding age group was subtracted from the measurements of dogs with CH. The plots suggest multimodal distribution of CFR in both groups and high variability among dogs with CH compared to the unaffected group.

Table S1 Breed and age of control dogs ≤6 months.

Table S2 Sex distribution.

Table S3 Influence of age: confidence intervals.
