# Supplementary figures and images for: Caudal Fossa Ratio in Normal Dogs and Eurasier Dogs with VLDLR-Associated Genetic Cerebellar Hypoplasia
Source: Front Vet Sci. 2018 Jan 22;4:241. doi: 10.3389/fvets.2017.00241 (PMC5786823; doi:10.3389/fvets.2017.00241)

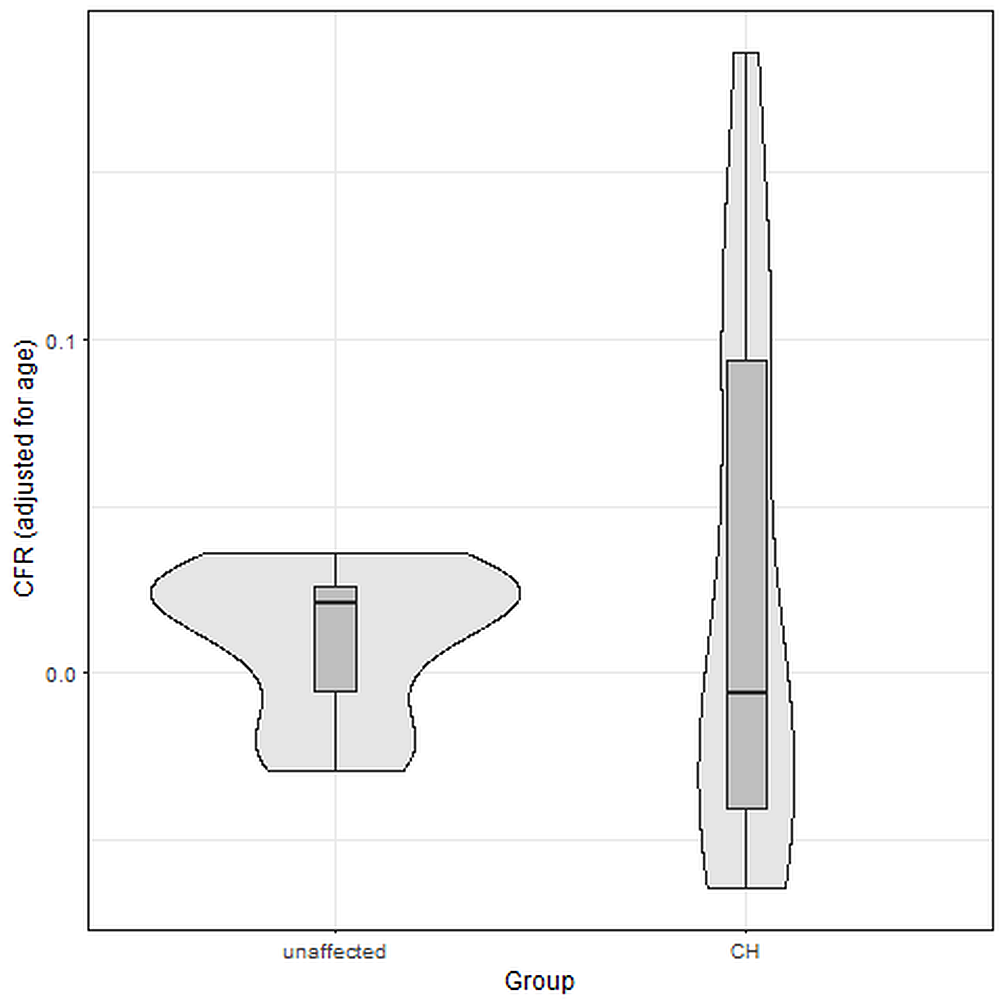

Supplement: Figure S1 — Exploratory data analysis of caudal fossa ratio (CFR) in Eurasier dogs. The plots depict the distribution of CFR in unaffected Eurasier dogs and Eurasier dogs with cerebellar hypoplasia (CH). To achieve better comparability, the CFR was adjusted for age. Measurements of unaffected dogs were centered according to age group. The mean of measurements of unaffected dogs from the corresponding age group was subtracted from the measurements of dogs with CH. The plots suggest multimodal distribution of CFR in both groups and high variability among dogs with CH compared to the unaffected group. [file image_1.tif]
