# Supplementary material for: Caudal Fossa Ratio in Normal Dogs and Eurasier Dogs with VLDLR-Associated Genetic Cerebellar Hypoplasia
Source: Front Vet Sci. 2018 Jan 22;4:241. doi: 10.3389/fvets.2017.00241 (PMC5786823; doi:10.3389/fvets.2017.00241)
Supplement: Table S1 — Breed and age of control dogs ≤6 months. [file table_1.docx]

| Breed | n | Age (month) |
| --- | --- | --- |
|  |  |  |
| Australian Shepherd | 1 | 3 |
| Black German Shepherd | 1 | 2 |
| Border Collie | 1 | 6 |
| Boxer | 1 | 6 |
| Chihuahua | 1 | 3 |
| Collie | 1 | 3 |
| Dachshund | 1 | 1 |
| English Setter | 1 | 1 |
| French Bulldog | 1 | 3 |
| German Shepherd | 3 | 2, 4, 4 |
| Jack Russel Terrier | 1 | 3 |
| Kromfohrländer | 3 | 1, 1, 1 |
| Labrador | 2 | 5, 6 |
| Magyar Vizsla | 1 | 5 |
| Miniature Dachshund | 1 | 4 |
| Mixed breed | 3 | 4, 4, 4 |
| Prague Ratter | 1 | 6 |
| Rhodesian Ridgeback | 1 | 5 |
| West Highland White Terrier | 1 | 1 |
| Unknown (not Eurasier) | 2 | 2, 5 |
| Total | 28 | 1 - 6 |
