# Supplementary material for: Caudal Fossa Ratio in Normal Dogs and Eurasier Dogs with VLDLR-Associated Genetic Cerebellar Hypoplasia
Source: Front Vet Sci. 2018 Jan 22;4:241. doi: 10.3389/fvets.2017.00241 (PMC5786823; doi:10.3389/fvets.2017.00241)
Supplement: Table S2 — Sex distribution. [file table_2.docx]

| Group | Total | Male | Female |
| --- | --- | --- | --- |
|  |  |  |  |
| Eurasier (affected) | 11 | 3 | 8 |
| Eurasier (unaffected) | 12 | 6 | 6 |
|  |  |  |  |
| Mesaticephalic | 47 | 29 | 18 |
| Australian Shepherd | 10 | 6 | 4 |
| Border Collie | 10 | 4 | 6 |
| Golden Retriever | 13 | 8 | 5 |
| Labrador | 14 | 11 | 3 |
|  |  |  |  |
| Brachycephalic | 24 | 12 | 12 |
| Boxer | 7 | 4 | 3 |
| French Bulldog | 10 | 2 | 8 |
| Pug | 7 | 6 | 1 |
|  |  |  |  |
| Puppies1-4 month | 20 | 11 | 9 |
| Puppies 5-6 month | 8 | 5 | 3 |
|  |  |  |  |
| CT vs. MRI group | 8 | 6 | 2 |
|  |  |  |  |
| Total no. dogs | 130 | 72 | 58 |
