# Supplementary material for: Caudal Fossa Ratio in Normal Dogs and Eurasier Dogs with VLDLR-Associated Genetic Cerebellar Hypoplasia
Source: Front Vet Sci. 2018 Jan 22;4:241. doi: 10.3389/fvets.2017.00241 (PMC5786823; doi:10.3389/fvets.2017.00241)
Supplement: Table S3 — Influence of age: confidence intervals. [file table_3.docx]

**Table S3**

|  | 2.5% | 97.5% |
| --- | --- | --- |
| (Intercept) | 0.25 | 0.26 |
| 5 - 6 mo. | 0.02 | 0.05 |
| 7 – 12 mo. | 0.02 | 0.05 |
| > 1y. | 0.03 | 0.05 |
